# A Wide-angle Multi-Octave Broadband Waveplate Based on Field Transformation Approach

**DOI:** 10.1038/srep17532

**Published:** 2015-12-07

**Authors:** Junming Zhao, Lianhong Zhang, Jensen Li, Yijun Feng, Amy Dyke, Sajad Haq, Yang Hao

**Affiliations:** 1 School of Electronic Engineering and Computer Science, Queen Mary University of London, London E1 4NS, United Kingdom; 2 School of Electronic Science and Engineering, Nanjing University, Nanjing, 210093, China; 3 School of Physics and Astronomy, University of Birmingham, Birmingham B15 2TT, United Kingdom; 4 Advanced Technology Centre, BAE Systems, Filton, FPC267, Bristol BS34 7QW.

## Abstract

Transformation optics (TO) offers a geometrical approach in designing optical components of any shapes. Although it has been proven to be a versatile and robust mathematical tool, TO has, however, limited control over electromagnetic (EM) field polarization in the process of coordinate transformation. Such a technique can be extended to a so-called “Field transformation (FT)” which provides *direct* control over the impedance and polarization signature of an arbitrary object. In this work, we demonstrate a FT application by designing and manufacturing a novel waveplate, which defies the fundamental limit of bandwidth and incident angles and has the ability of converting between TE (transverse electric) and TM (transverse magnetic) as well as LCP (left-handed circular polarization) and RCP (right-handed circular polarization). Such a waveplate can also be applied to different operating modes for both transmitted and reflected waves by adjusting its thickness and adding an optional metallic ground plane. The proposed design approach presents a remarkable degree of advance for designing future devices with arbitrary polarization controls, artificial waveguides or antenna substrates and polarization-enabled resonators with angle-insensitive functionalities. Our approach has far reaching implications applicable from radio to optical frequencies.

Controlling polarization states is one of the most important topics in manipulating electromagnetic (EM) waves. In the present state of the art, various physical mechanisms, such as those used in circular dichroism of chiral media^1^,^2^, birefringence of anisotropic materials^3^,^4^ and Brewster effects^5^,^6^ from both natural and artificial structures have been explored. Consequently, various devices have been proposed for polarization conversions between TE and TM^3^,^4^,^7^,^8^,^9^, LCP and RCP^10^, linear and circular waves^11^,^12^,^13^,^14^. However, existing designs are only adapted to either normal or a specific angle of incidence with a fundamental limit for the gain-bandwidth product. There have been many attempts recently to develop new theories and technologies to overcome those limits. Among them, the technique of transformation optics (TO) is especially notable and has been applied to the design of optical components with extraordinary properties^15^,^16^,^17^. Although it has been proven to be a versatile and robust mathematical tool, TO requires a “virtual” space/design to start with and often has no control over electromagnetic (EM) field polarization in the process of coordinate transformation. Instead, a new approach utilizing field transformation (FT) was recently developed to achieve dynamic control and manipulation of the polarization state of EM waves as a complementary technique to TO, and as an approach to generate pseudo magnetic field in bending light^18^,^19^.

In this work, we demonstrate a remarkable degree of fundamental and conceptual advances by applying the FT technique in the design of waveplates utilizing artificial anisotropic dielectrics. The study of waveplates can be dated back to 1960s, when it was used to increase angular resolution of ultrasonic scanning for medical imaging^20^. The applications for waveplates in optics have been steadily increasing thanks to the rapid development of nanotechnologies. While previous designs often use birefringent crystalline materials, polymers and liquid crystals, there have been a suite of devices demonstrated by using artificial materials such as photonic crystals^21^, negative refractive index metamaterials^22^ and more notably, metasurfaces by nanoimprint lithography^23^. These designs, which are usually narrowband, have poor transmissions and operate at limited incident angles, and, they can find practical uses such as power attenuation of a laser, optical isolation and polarized light control. In some other applications, for example, the astronomical polarimetry, it requires the waveplate to function in the wavelength range of 200–600 μm and a wide angular range at room and cryogenic temperatures^24^,^25^. This has been a long-standing challenge in measurements of the Cosmic Microwave Background (CMB) temperature anisotropies^26^. In^27^, a design tool based on optimization techniques has been proposed to develop a quarter-waveplate with wideband and wide viewing angle for three-dimensional liquid crystal display. We argue that a generic mathematical tool is urgently needed based on classic Maxwell’s Equations by linking polarized field components with constitutive material properties and their surface impedances of resulting media. Just like many studies emerged in the application of TO, we will show the power of field transformation in the optimal design of EM devices such as a waveplate, that are required to be broadband and independent of incident angle, bearing in mind that current waveplates not only are cumbered by the laws of physics but also cost ranging from tens to thousands of dollars.

In this paper, we will firstly lay down the design approach based on field transformation and demonstrate that by carefully choosing effective material properties, a half-waveplate is manufactured for broadband operation with a total conversion efficiency of more than 90% and an angular range up to 60°, comparing with those at the normal incidence. Such a waveplate can also be applied to different operating modes for both transmitted and reflected waves by adjusting its thickness and adding an optional metallic ground plane. We prove through measurements that the proposed technique has remarkable advantages over other existing schemes and can be further explored to overcome the fundamental limit of other EM devices such as high impedance surfaces (HIS) or artificial perfect magnetic conductors (PMC), and can be applied to a broad range of applications from microwave to optics.

## Results

### Design and operation of field transformation based waveplate

We start with a 2D case of in-plane wave propagating on the *x-y* plane and assume that the virtual space before FT is vacuum with decoupled wave propagations for TE (transverse electric, *E*_*z*_-field) and TM (transverse magnetic, *H*_*z*_-field) polarizations separately^18^. The fields and material parameters in the virtual space are denoted with a superscript “(0)”. The applied FT is defined by
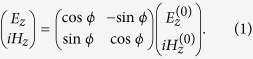


In Eq. (1), a nonzero 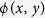 induces additional off-diagonal terms between the transverse and the *z* components in permittivity and permeability tensors as


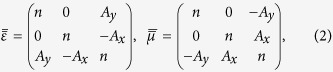


where 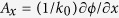, 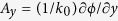 where *k*_0_ is the wavenumber of the vacuum. These additional off-diagonal terms enable the coupling between the components of TE and TM polarizations, which is the primary cause of the polarization conversion. The definitions and the derivations of the FT are given in more detail in the supplementary material and reference^18^.

**Figure 1:**
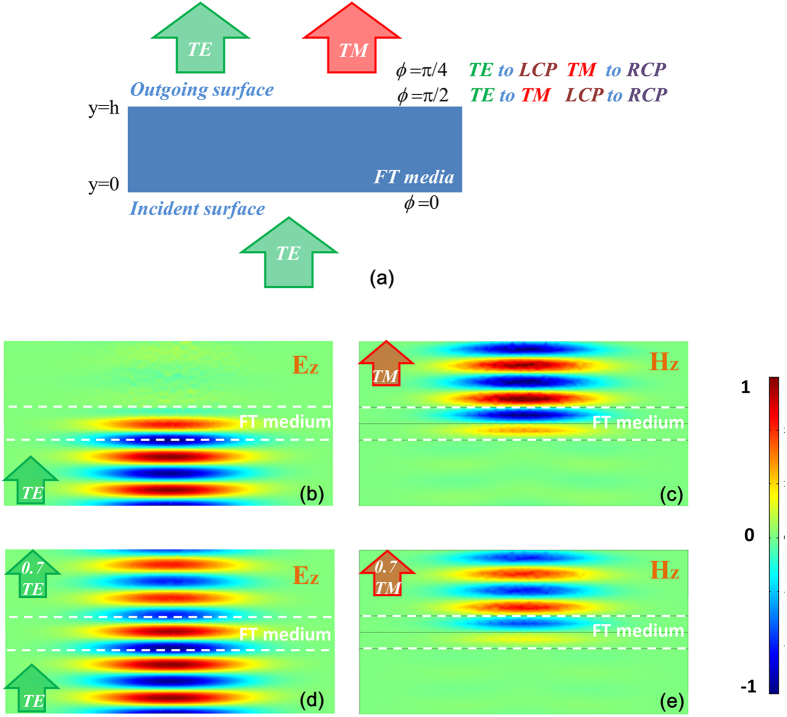
(**a**) Schematic diagram of the proposed FT transmitted waveplate. (**b**,**c**) Normalized field distributions for totally TE to TM transmitted conversion through Comsol. (**d**,**e**) Results for partially TE to TM conversion resulting linear to circular polarization conversion.

Suppose we have a device from 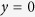 to 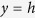 with 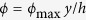, thus 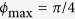 and 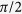 correspond to a quarter-waveplate and a half-waveplate as shown in Fig. 1(a). This is also the simplest form of field transformation as the obtained medium is homogeneous since we have utilized a function of 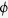 linear with *y*. The medium parameters become 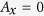, 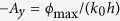. It is worth noting that the waveplate design obtained here is independent of incidence angle.

If we apply *ϕ*_max_ = *π*/2, the FT medium now acts as a polarization converter from TE to TM with −*π*/2 phase shift, operating as a half-waveplate. Its thickness is chosen to be one wavelength in order to achieve required phase shift. Numerical simulation results (Comsol Multiphysics, at normal incidence as an example) of the waveplate are shown in Fig. 1(b,c). As shown in the figures, an incident TE Gaussian beam has been completely converted into a TM beam. Meanwhile, for the circularly polarized incident wave, a phase flip of 180 degrees is introduced into two orthogonal linear components, which lead to a total transmitted transmission for conversion to cross-polarization, the polarized wave with opposite handness. If we apply *ϕ*_max_ = *π*/4 to the FT medium, for an incident TE wave, the power can be equally divided into TE and TM modes. The transmitted TM mode has −*π*/2 phase delay with respect to the transmitted TE mode, which lead to an outgoing circular polarized wave. Thus far, a linear to circular polarization converter is achieved, which acts as a quarter-waveplate as demonstrated in Fig. 1(d,e).

As the off-diagonal term *A*_*y*_ is inverse proportional with *h,* the thickness of the FT medium, both the half-waveplate and quarter-waveplate can be obtained by adjusting the thickness of the same FT medium instead of applying different 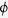 values. Thus if we properly design a TE to TM polarization converter utilizing one FT medium with a thickness of *h*, the one with a thickness of *h*/*2* utilizing the same medium with same off-diagonal terms can behave as a linear to circular polarization converter.

**Figure 2:**
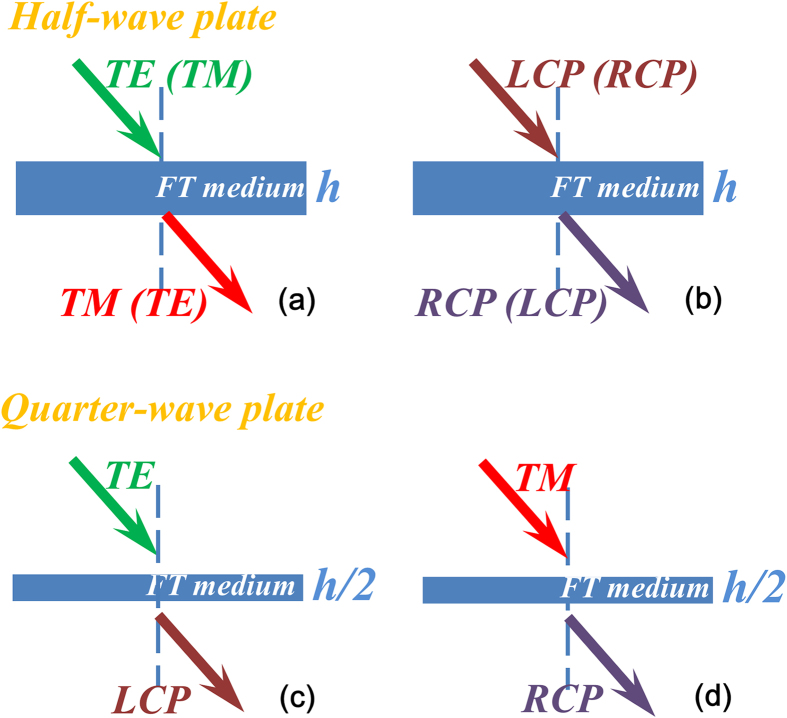
Different modes of operation of the FT medium.

Indeed, such a FT medium can also achieve various other operational functions for polarization control. Figure 2 summarizes the different operation modes. (a) and (b) are the half-waveplates in converting TE to TM or LCP to RCP (and vice and versa). (c) and (d) are the quarter-waveplates in transmission mode, which can be used to convert linear to circular polarization. In addition, by introducing a metallic backplane to such a waveplate, further polarized wave conversions can be extended to reflected waves, and details of such a scheme can be found in the Supplementary Information.

### Realisation of field transformation based waveplate

One of the challenges for realizing such waveplates is to obtain a medium with off-diagonal terms in both permittivity and permeability as described in Eq. (2). It is non-trivial to acquire an impedance matching medium with equal permittivity and permeability values. A mathematical approximation has been made to eliminate the requirement of magnetic materials in the design of waveplate. Such a modification can be achieved based on secular equation (see Supplementary Information) as shown below:


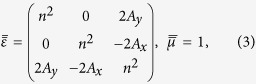


**Figure 3:**
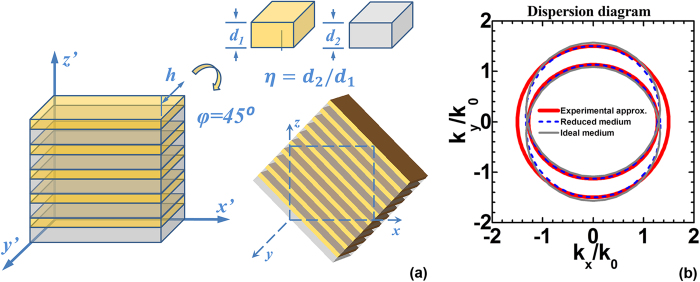
(**a**) Schematic diagram of the realization of the FT waveplate. (**b**) The dispersion surfaces of the ideal medium and the experimental approximated one.

Comparing Eq. (3) with Eq. (2), values of the off-diagonal permittivity have been doubled to meet the constitutive parameter requirements for the waveplate. Instead of adopting a metasurface approach, we propose to realize the waveplate by rotating an artificially designed material slab with fine dielectric gratings around the origin of the coordinates in *x*′*-z*′ plane clockwise by 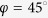 to *x*-*z*. The required anisotropic material with corresponding permittivities ε_*x’ ,*_ε_*y’*_ and ε_*z’*_for the FT wave-plate can be artificially designed using a set of two alternating dielectrics with permittivities of ε_*1*_ and ε_*2*_ based on the effective media theory^28^, 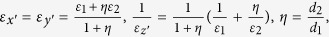
where *d*_*1*_/*d*_*2*_ is the layer thickness to be properly chosen and both *d*_*1*_ and *d*_*2*_ are much smaller than the wavelength of operation. The geometrical schematic diagram is shown in the Fig. 3(a). The dispersion curves of all the media including medium of ideal constitutive material parameters from Eq. (2), the parameter reduced medium and the proposed artificial anisotropic dielectrics with aforementioned approximations are plotted in Fig. 3(b) by applying 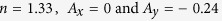. Our medium approximates the dispersion properties to those of an ideal medium required from theoretical calculations from the field transformation (FT) for a large range of angles.

### Experimental demonstration of FT-based waveplate

**Figure 4:**
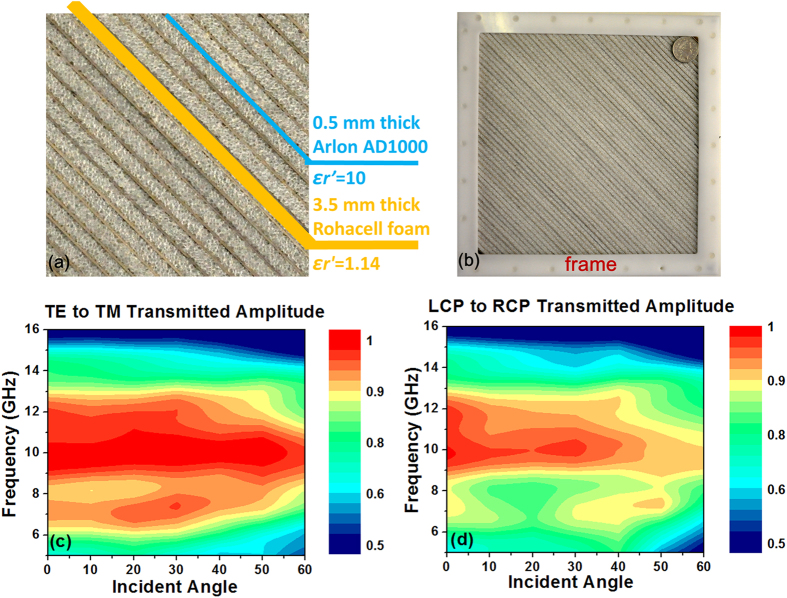
Fabricated FT waveplate in microwave regime, (**a**) zoom-in photo of the alternating dielectric layers, (**b**) photo of the sample and measured results of (**c**) TE to TM conversion amplitude (**d**) LCP to RCP conversion amplitude.

To validate the design approach presented in this paper, a half-waveplate for TE to TM transmitted conversion is fabricated and measured at microwave frequencies without loss of its generality. The fabricated sample consists of ultra-thin dielectric gratings with alternating materials having respective high and low permittivities (Fig. 4(a)). The high dielectric material is Arlon AD1000 with a permittivity value of 10+/−0.35 and a loss tangent of 0.0023, and the low dielectric material is Rohacell foam having a permittivity of 1.14-0.01*i*. The materials are contained in a frame arrangement. The frame is manufactured from Delrin®, a polyoxymethylene based polymer material known for its high strength, stiffness and dimensional stability (Fig. 4(b)). The frame consists of a rectangular piece with sawtooth edges that have been machined with dimensions to allow precise location of strips of the high permittivity and low permittivity layers. The frame is fabricated from four straight sections that are constructed into a square arrangement. Two flat pieces to prevent movement of the layers are screwed on to the top and bottom surfaces using nylon screws to leave an exposed measurement area of the layered materials with an area of 202 mm^2^. The sawtooth edges have a pitch of 4 mm and are angled at 45°, with a 4 mm slot length. The dimensions were selected to accommodate the Rohacell foam and the Arlon material, of thickness 0.5 mm and 3.5 mm respectively. The frame without the flat securing plates is of 30 mm thickness, and the plates are each of 2 mm thickness. The whole sample is shown in Fig. 4(b). The measurement is carried out utilizing a pair of double-ridged guide antennas in a mini chamber connected to an Agilent vector network analyzer. The results are shown in Fig. 4 (c,d). As we can see in the figure, for TE to TM conversion, nearly 80% 3 dB bandwidth is achieved (6–14 GHz) for a center frequency of 10 GHz with incident angles up to 50°. For the incident angle of 60°, a 3 dB bandwidth over 7–14 GHz is still achieved. The conversion amplitude is over 90% across 8–13 GHz with incident angles up to 50°.

**Figure 5:**
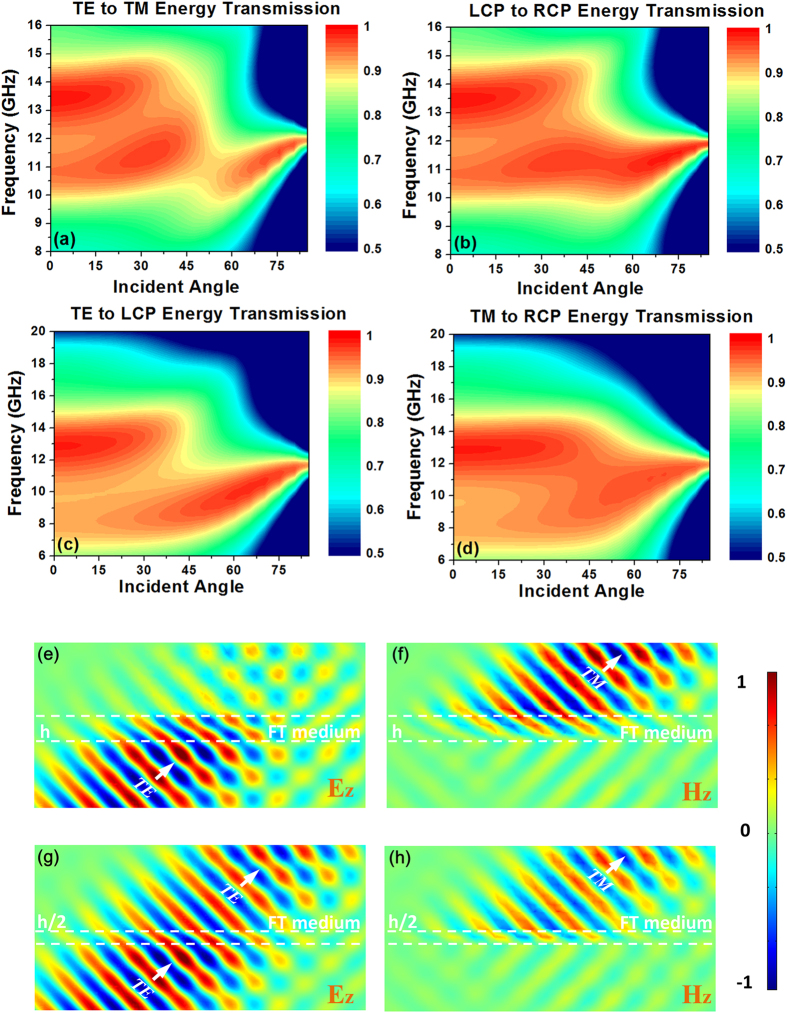
Full wave simulation results (**a**) TE to TM energy transmission, (**b**) LCP to RCP energy transmission, (**c**) TE to LCP energy transmission, (**d**) TM to RCP energy transmission, through CST; (**e**) TE to TM transmission electric field pattern, (**f**) TE to TM transmission magnetic field pattern, (**g**) TE to LCP transmission electric field pattern, (**h**) TE to LCP transmission magnetic field pattern, at incident angle of 45° through Comsol.

Full-wave simulations for the physical dielectric grating structure through both CST and Comsol have also been carried out to verify the performance of such a waveplate shown in the Fig. 5. Conversion efficiencies correspond to different operational modes illustrated in Fig. 2 are shown in Fig. 5(a–d). As can be seen from the figures, the center frequency has slightly shifted to a higher frequency and is mainly due to the approximation procedure used which induces large diagonal terms simultaneously with off-diagonal terms in the permittivity tensors. By removing anisotropic magnetic terms, a slight impedance mismatch with free space results. Despite this, the waveplate still maintains a conversion efficiency of over more than 90% between 8–12 GHz with the incident angle up to 45°. For incident angles up to 60°, the energy conversion efficiency remains over 80% in the same frequency range. The field patterns of TE to TM and TE to LCP for an incident angle of 45° are shown in Fig. 5(e–h), which clearly demonstrate the functionality of proposed waveplate for wide angle and broadband polarization conversion. To demonstrate the excellent performance of our waveplate, a table (Table 1) is provided comparing the efficiency and other performance of our sample with the earlier reported polarizers in the references. Our proposed waveplate exhibits great advantages comparing with the earlier reported designs.

## Discussion

As we can see in both simulation and measurement results, due to the approximation from a pure dielectric implementation, mismatch in the wave impedance causes a small reduction in the conversion efficiency and operating bandwidth in comparison with those from theoretical calculations. When the incident angle increases beyond 60°, the propagation distance in the experimentally approximated FT media gradually changes and the phase retardation of different polarized components will deviate from the designed values. Thus it affects the performance of the waveplates. Such influences become more obvious for large incident angles when the frequency changes. Hence, the bandwidths of waveplates become narrower when the incident angles are increased. Comparing with the simulation results, the conversion efficiency in experiment is lower especially for large incident angle. This is mainly caused by the parameter variation in the real fabrication, including the slight change of the permittivity, permeability and the layer dimensions. However the proposed structure made of fine dielectric gratings possesses both broad bandwidth and wide angular resolution than conventional designs. The conversion efficiency can reach up to 90% and only reduces by less than 10% at an angle of 60°, comparing with those at the normal incidence. In principle, it can be further improved by using a sample with increased thickness, which leads to a smaller *A*_*y*_, so that the effective medium approximation can be made more accurate. However, the best possible solution is to use magneto-dielectrics in the design to ensure the surface impedance is well matched to free space.

In conclusion, we have designed a wide-angle broadband waveplate through the FT approach, which is realized through sub-wavelength dielectric gratings based on effective media theory. Such waveplate can be easily extended to multiple applications by adjusting its thickness, achieving the polarization conversion of incident waves from linear to linear, circular to circular, and linear to circular polarizations respectively. The performance of such waveplate has been verified through both analytical and numerical simulations with measurements carried out in the microwave regime. The proposed design technique is unique and can be applied to manipulate all kind of waves including EM waves from radio to optical frequencies.

## Methods

### Experiemental setup

**Figure 6:**
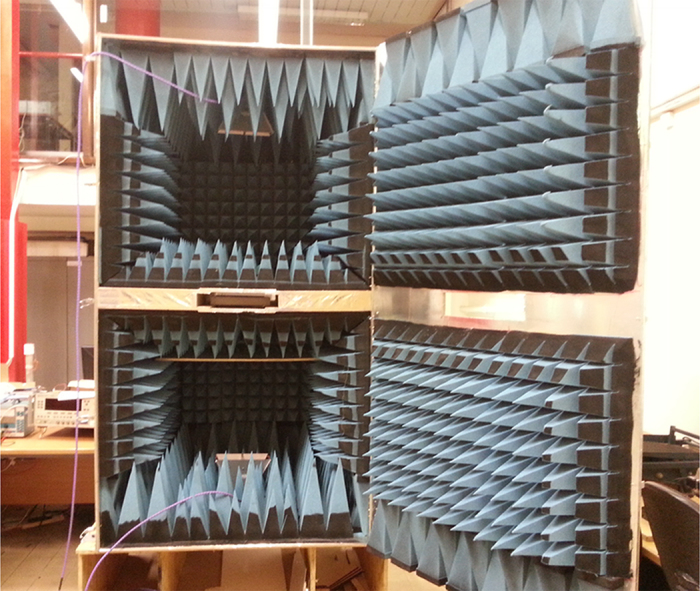
Photo of the experimental measurement setup.

The measurement was carried out using a purpose-built chamber as shown in the Fig. 6. A pair of double-ridged guide antennas (3115 Double-Ridged Guide Antenna from ETS-Lindgren) is fixed to the top and bottom part of the chamber separately. By connecting them to an Agilent vector network analyzer with cables the transmission coefficient can be measured. For the measurement of oblique incident angles, we rotate the sample with respect to the horizontal plane. The bottom of the anechoic chamber is set as the reference plane and the sample under test is aligned with the reference plane. Different incident angles are set up by adjusting the distance between the other edge of the sample and the reference plane with premade markers.

### Numerical simulation

Numerical simulations including designs from both the analytical model and the effective medium approximation are performed using the COMSOL 3D RF module. The final design of physically realized structures consisting of multilayer dielectric composites is performed by full wave simulation through CST microwave studio.

## Additional Information

**How to cite this article**: Zhao, J. *et al.* A Wide-angle Multi-Octave Broadband Waveplate Based on Field Transformation Approach. *Sci. Rep.* **5**, 17532; doi: 10.1038/srep17532 (2015).

## Supplementary Material

Supplementary Information

## Figures and Tables

**Table 1:** Performance comparison of earlier reported designs in the references with our design.

Material type	Operation mode	Bandwidth (−3DB)	Peak Efficiency	Incident angle	Reference
Silver grating	Reflective Half-waveplate	Over 70% (optical 600 nm)	90%	Normal incidence	4
Metallic grating	Reflective Half-waveplate	57% (14 GHz)	95%	11.5°	7
Anisotropic Metamaterial	Reflective Half-waveplate	12% (13.5 GHz)	95% (80% for 45°)	0–45°	8
Chiral Metamaterial	Transmitted Half-waveplate	8% (12.5GHz)	97%	Normal incidence	9
Chiral Metamaterial	Transmitted Half-waveplate	5% (4.8GHz)	27.5%	Normal incidence	10
Copper wire grids sheets	Transmitted Quarter-waveplate	46%	90%	Normal incidence	11
Metal grids sheets	Transmitted Quarter-waveplate	Over 21% (95 GHz)	94%	5°	12
Impedance surfaces	Reflective Quarter-waveplate	Over 40% (13 GHz)	95% for 0⁰	0–45°	13
Meta-surface	Transmitted Quarter-waveplate	8% (2.4 GHz)	90%	Normal incidence	14
Our work	Both reflective and transmitted for both quarter and half waveplate	80% (10 GHz)	98%	0–60%	
