# Supplementary material for: A Wide-angle Multi-Octave Broadband Waveplate Based on Field Transformation Approach
Source: Sci Rep. 2015 Dec 7;5:17532. doi: 10.1038/srep17532 (PMC4671019; doi:10.1038/srep17532)
Supplement: Supplementary Information [file srep17532-s1.pdf]

Supplementary Information

A Wide-angle Multi-Octave Broadband Waveplate Based on Field Transformation Approach

Junming Zhao[1,2], Lianhong Zhang[1], Jensen Li[3], Yijun Feng[2],
Amy Dyke[4], Sajad Haq[4] and Yang Hao[1,*]

[1]School of Electronic Engineering and Computer Science, Queen Mary University of London, London E1 4NS, United Kingdom.

[2]School of Electronic Science and Engineering, Nanjing University, Nanjing, 210093, China.

[3]School of Physics and Astronomy, University of Birmingham, Birmingham B15 2TT, United Kingdom.

[4]Advanced Technology Centre, BAE Systems, Filton, FPC267, Bristol BS34 7QW.

Reduced parameters of FT medium. In this supplemental material, we specify the dispersion equation of the eigenmodes of the FT media and the reduced parameters cases. For the 2D in-plane wave propagations on the x-y plane, Maxwell's equations (in Heaviside-Lorentz units) can then be written as

$$\nabla \times \mu_{TT}^{-1} \cdot \nabla \times \hat{z}E_z = k_0^2 \varepsilon_{zz} \hat{z}E_z,$$
$$\nabla \times \varepsilon_{TT}^{-1} \cdot \nabla \times \hat{z}H_z = k_0^2 \mu_{zz} \hat{z}H_z,$$

where $\mu_{TT} / \varepsilon_{TT}$ is the 2×2 symmetric tensor for the transverse permittivity or permeability in the x and y directions and k_0 is the wave number in vacuum.

As we have derived

$$\overline{\overline{\varepsilon}}=\begin{pmatrix} n & 0 & A_y \\ 0 & n & -A_x \\ A_y & -A_x & n \end{pmatrix},\quad \overline{\overline{\mu}}=\begin{pmatrix} n & 0 & -A_y \\ 0 & n & A_x \\ -A_y & A_x & n \end{pmatrix},$$

the dispersion surface/secular equation of the eigenmodes of the FT media can be written as

$$Det[\begin{pmatrix} ik_x & ik_y & 0 & 0 \\ 0 & 0 & ik_x & ik_x \end{pmatrix}\cdot(-c\cdot\begin{pmatrix} ik_x & 0 \\ ik_y & 0 \\ 0 & ik_x \\ 0 & ik_y \end{pmatrix}-\alpha)+\beta\cdot\begin{pmatrix} ik_x & 0 \\ ik_y & 0 \\ 0 & ik_x \\ 0 & ik_y \end{pmatrix}+a]=0,$$

where

$$c=\begin{pmatrix} \frac{1}{n} & 0 & 0 & 0 \\ 0 & \frac{1}{n} & 0 & 0 \\ 0 & 0 & \frac{1}{n} & 0 \\ 0 & 0 & 0 & \frac{1}{n} \end{pmatrix},\qquad \alpha=\begin{pmatrix} 0 & \frac{A_x k_0}{n} \\ 0 & \frac{A_y k_0}{n} \\ -\frac{A_x k_0}{n} & 0 \\ -\frac{A_y k_0}{n} & 0 \end{pmatrix},\qquad \beta=\begin{pmatrix} 0 & 0 & -\frac{A_x k_0}{n} & -\frac{A_y k_0}{n} \\ \frac{A_x k_0}{n} & \frac{A_y k_0}{n} & 0 & 0 \end{pmatrix},$$

$$a=\begin{pmatrix} \frac{k_0^2(A_x^2+A_y^2-n^2)}{n} & 0 \\ 0 & \frac{k_0^2(A_x^2+A_y^2-n^2)}{n} \end{pmatrix}.$$

Then, we can lump all the magnetic response into the permittivity by keeping a similar shape of dispersion surface. This is in the similar spirit of the so called reduced-parameter approximation in transformation optics to make fabrication easier [*Science* **312**, 1780 (2006) by Pendry and Smith]. Then the approximated medium is

$$\overline{\overline{\varepsilon}}=\begin{pmatrix} n_1 & 0 & 2A_y\sqrt{n_1} \\ 0 & n_1 & -2A_x\sqrt{n_1} \\ 2A_y\sqrt{n_1} & -2A_x\sqrt{n_1} & n_1 \end{pmatrix},\quad \overline{\overline{\mu}}=1.$$

$$n_1=A_x{}^2+A_y{}^2+n^2$$

Further approximation on the above medium gives the medium with the reduced medium in text as

$$\overline{\overline{\varepsilon}} = \begin{pmatrix} n^2 & 0 & 2A_y \\ 0 & n^2 & -2A_x \\ 2A_y & -2A_x & n^2 \end{pmatrix}, \quad \overline{\overline{\mu}} = 1.$$

In this work, we further realize this reduced medium (with $n = 1.33$ and $A_x = 0,\ A_y = -0.24$) except the component ε_{yy} by stacking layers of alternating dielectric materials. The experimentally realized medium can then be specified by the following tensors:

$$\overline{\overline{\varepsilon}} = \begin{pmatrix} n^2 & 0 & 2A_y \\ 0 & \varepsilon_y & 0 \\ 2A_y & 0 & n^2 \end{pmatrix} = \begin{pmatrix} 1.76474 & 0 & -0.48276 \\ 0 & 2.2475 & 0 \\ -0.48276 & 0 & 1.76474 \end{pmatrix}, \quad \overline{\overline{\mu}} = 1$$

Reflection mode. We also find that the proposed linear to circular polarization converter (quarter-waveplate) in transmitted mode has the same constitutional parameters with the PC2 reflective mode in [18]. That implies that we obtain a TE to TM reflective converter by just putting an *h/2* waveplate over a PEC. Meanwhile it can be proved that such an *h/2* PC2 can reflect a circularly polarized wave to its co-polarization instead of cross-polarization for conventional PEC. Besides, for the FT medium with a thickness of *h/4* on PEC, it exhibits to be a reflective converter between linear and circular polarizations. At last, by putting the original TE to TM transmitted converter with thickness of *h* on PEC, we obtain a mimicking PMC surface by applying extra small *h* value. All the expanded modes are summarized in Fig. 1. In particular, all the cases are independent of the incident angle in the *x*-*y* plane. The simulated energy reflection ratios for different cases are shown in Fig. 2.

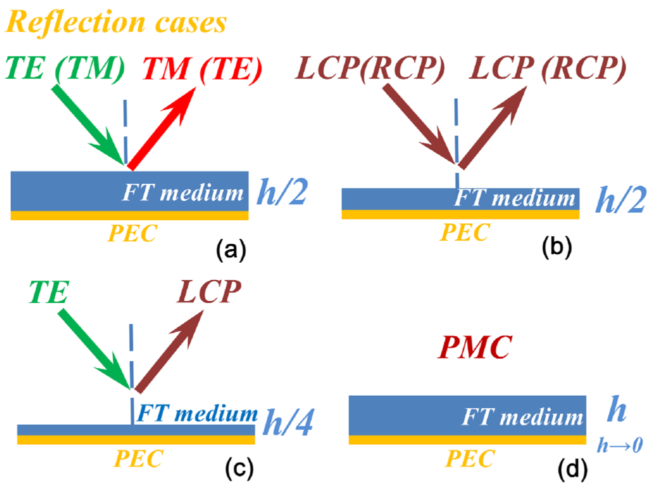

Figure 1 | Different reflective modes of operation of the FT medium.

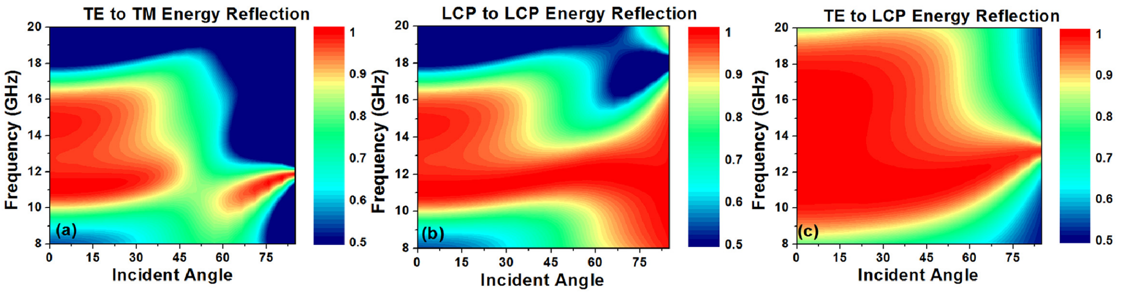

Figure 2 | Simulated energy conversion ratios for different reflection modes. (a) TE to TM (b) LCP to LCP (c) TE to LCP.

Axial ratio. Here we give the simulated axial ratio result for Figs. 4(d) and 5(d) of the main text in Fig. 3. It exhibits a broadband 3dB bandwidth, which verifies the converted RCP with a good circular polarization.

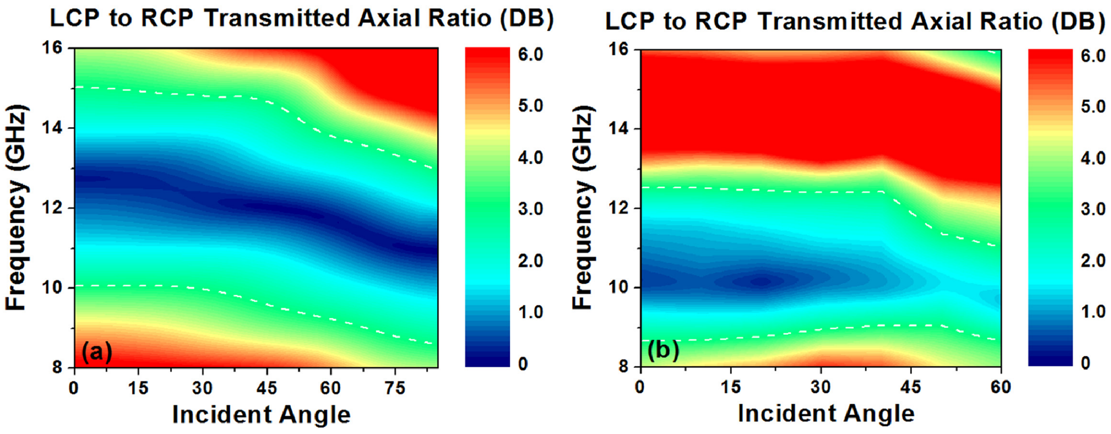

Figure 3 | LCP to RCP conversion axial ratio (a) Simulated result and (b) measured result.
